# Supplementary material for: Convergent coexpression reveals shared biological mechanisms underlying common and rare variant risk in six neuropsychiatric disorders
Source: Mol Psychiatry. 2026 Apr 7;31(8):4787–98. doi: 10.1038/s41380-026-03571-x (PMC13364661; doi:10.1038/s41380-026-03571-x)
Supplement: Supplementary file 1 — Supplementary figures [file 41380_2026_3571_MOESM1_ESM.docx]

**Supplementary figures**

Figure S1. Overlap of prioritized GWAS genes across different approaches for six brain disorders

Figure S2. Convergence similarity across the 13 brain tissues from CMC and GTEx

Figure S3. **Comparison of convergence Z-score distributions across GWAS gene prioritization methods**

Figure S4. Distribution of GWAS and burden convergent coexpression Zscores across 13 brain tissues from GTEx and CMC

Figure S5. Excess overlap for essential disease genesets across

Figure S6. Correlation of convergence with disease association pvalue

Figure S7. Stratification of Genes by Direction of Convergence Z-Scores

Figure S8. Median constraint level distribution for convergent genes

Figure S9. Excess overlap of known drug target genes at different clinical phases in convergent genes

Figure S10. Enrichment of known rare variant burden and GWAS disease risk genes in top positive and negative convergent genes


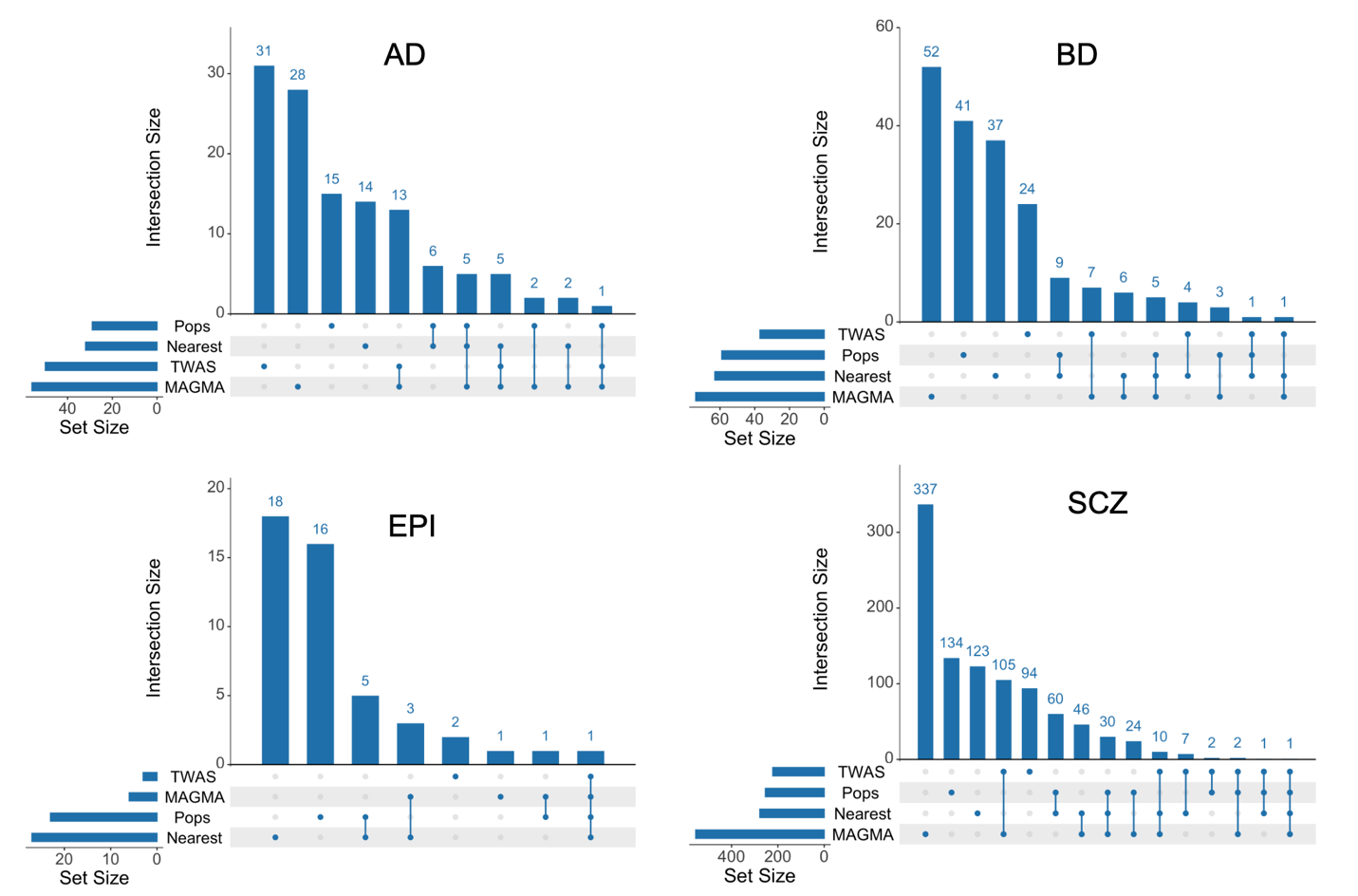


**Figure S1.** Upset plots showing overlap of prioritized GWAS genes across different approaches (TWAS, MAGMA, PoPS, and Nearest gene).


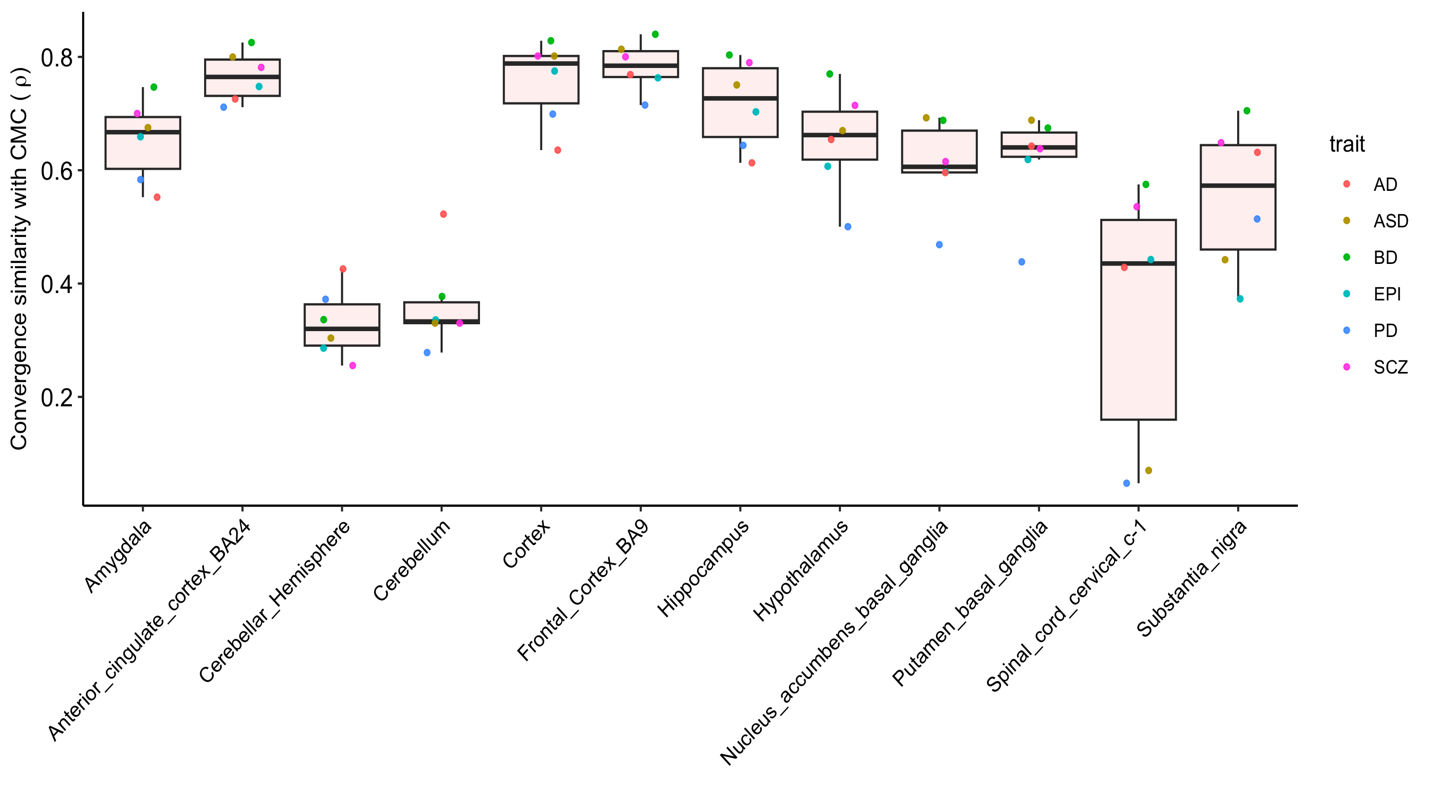


**Figure S2.** Similarity of burden convergence Z-score measured by spearman’s rho between that of CMC and 12 brain tissues from GTEx .


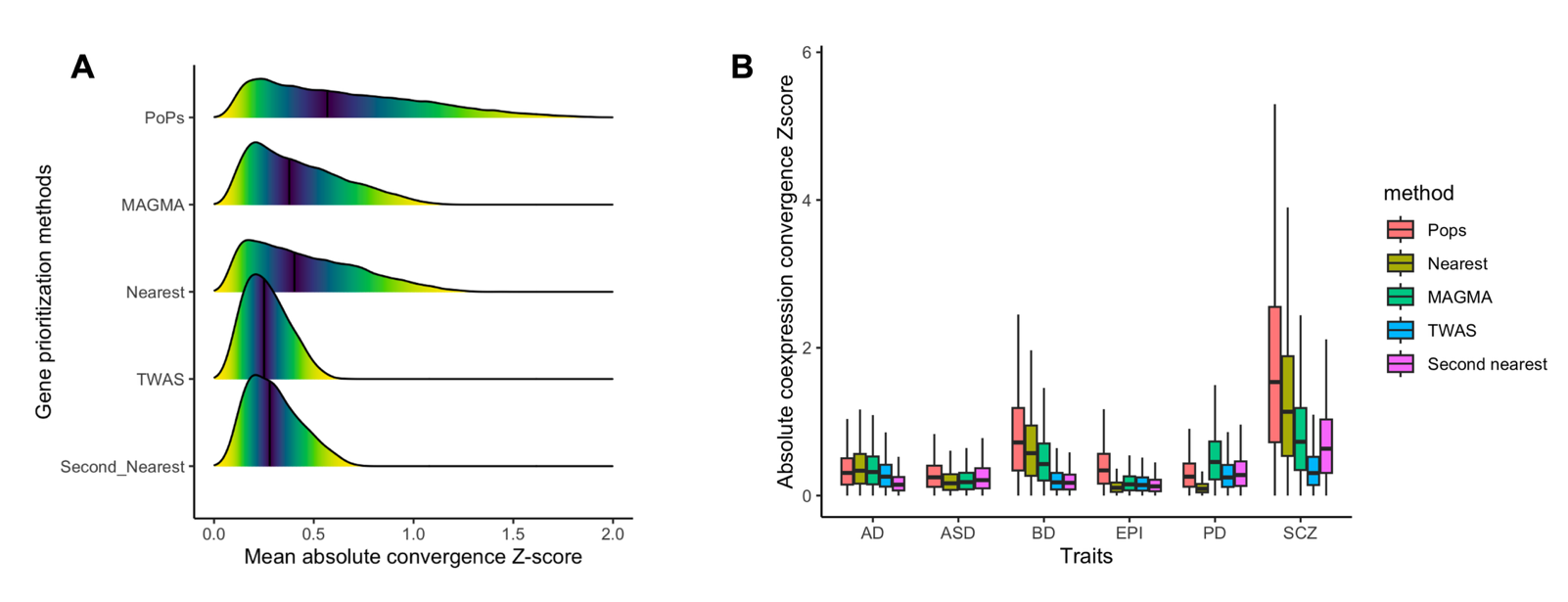


**Figure S3.** **Comparison of convergence Z-score distributions across GWAS gene prioritization methods.** A) Distribution of mean absolute convergence Z-scores across five prioritization methods. B) Distribution of mean absolute convergence Z-scores across five methods, stratified by disorder


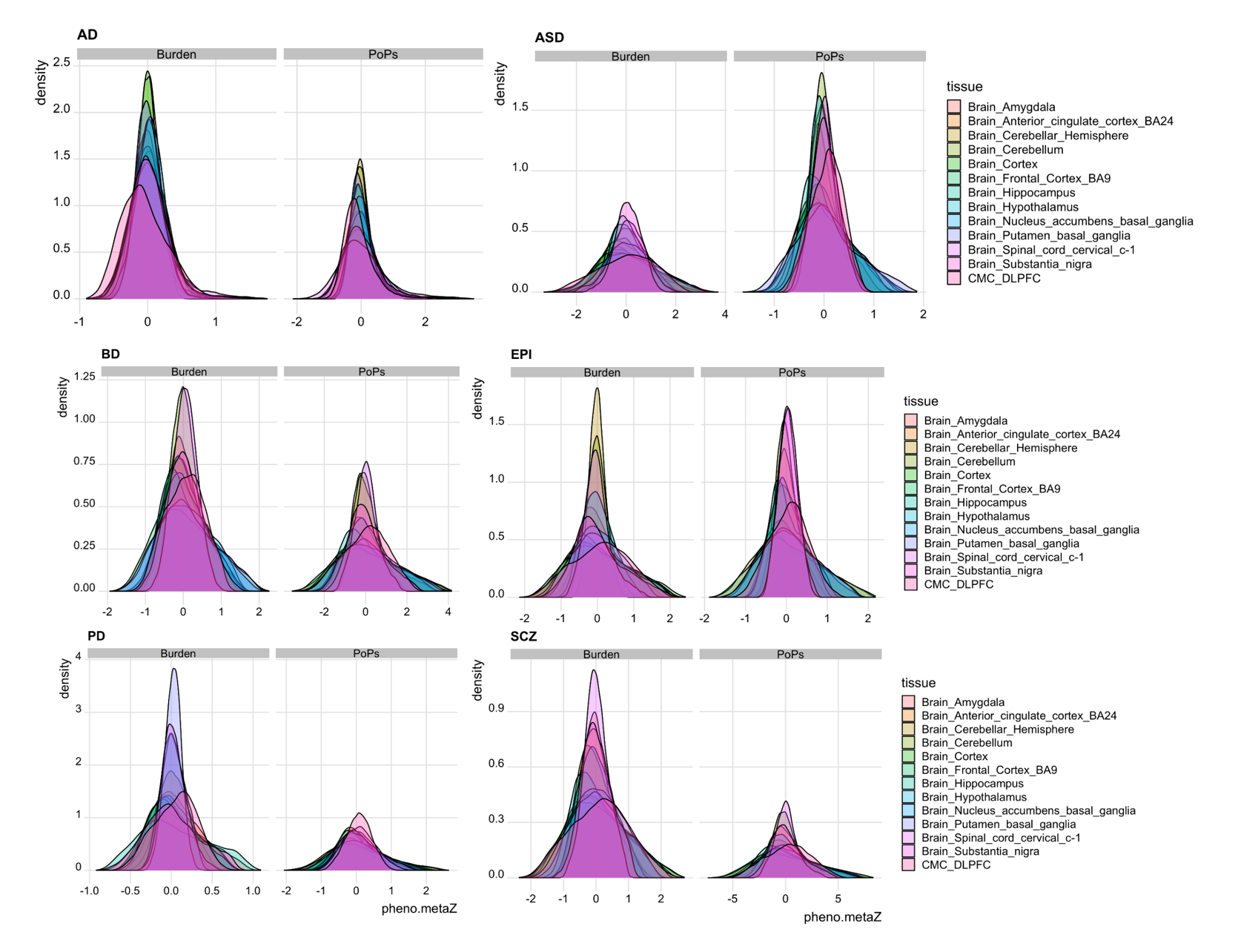


**Figure S4.** Distribution of GWAS (prioritized by PoPS) and rare variant burden convergent coexpression Zscores across 13 brain tissues from GTEx and CMC for the six brain disorders


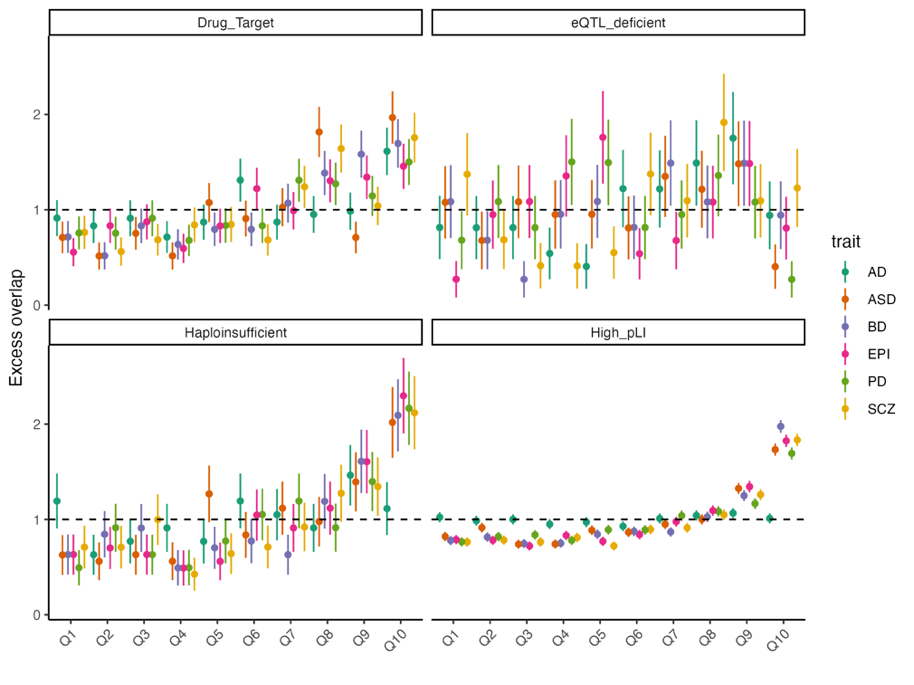


GWAS convergence


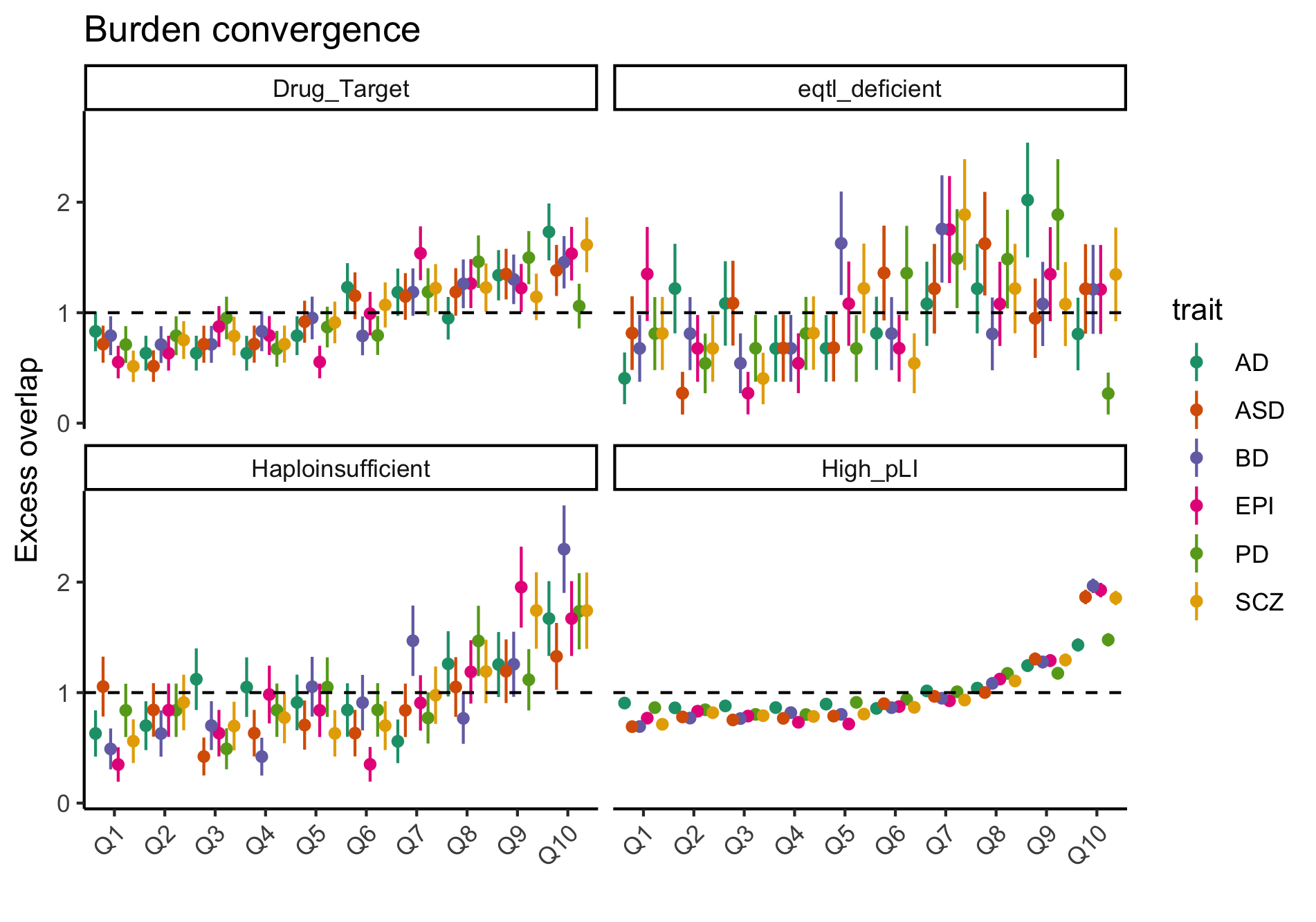


**Figure S5.** Excess overlap for essential disease genesets in both GWAS and rare variant burden convergence stratified by percentile of absolute convergence Zscore for the six disorders


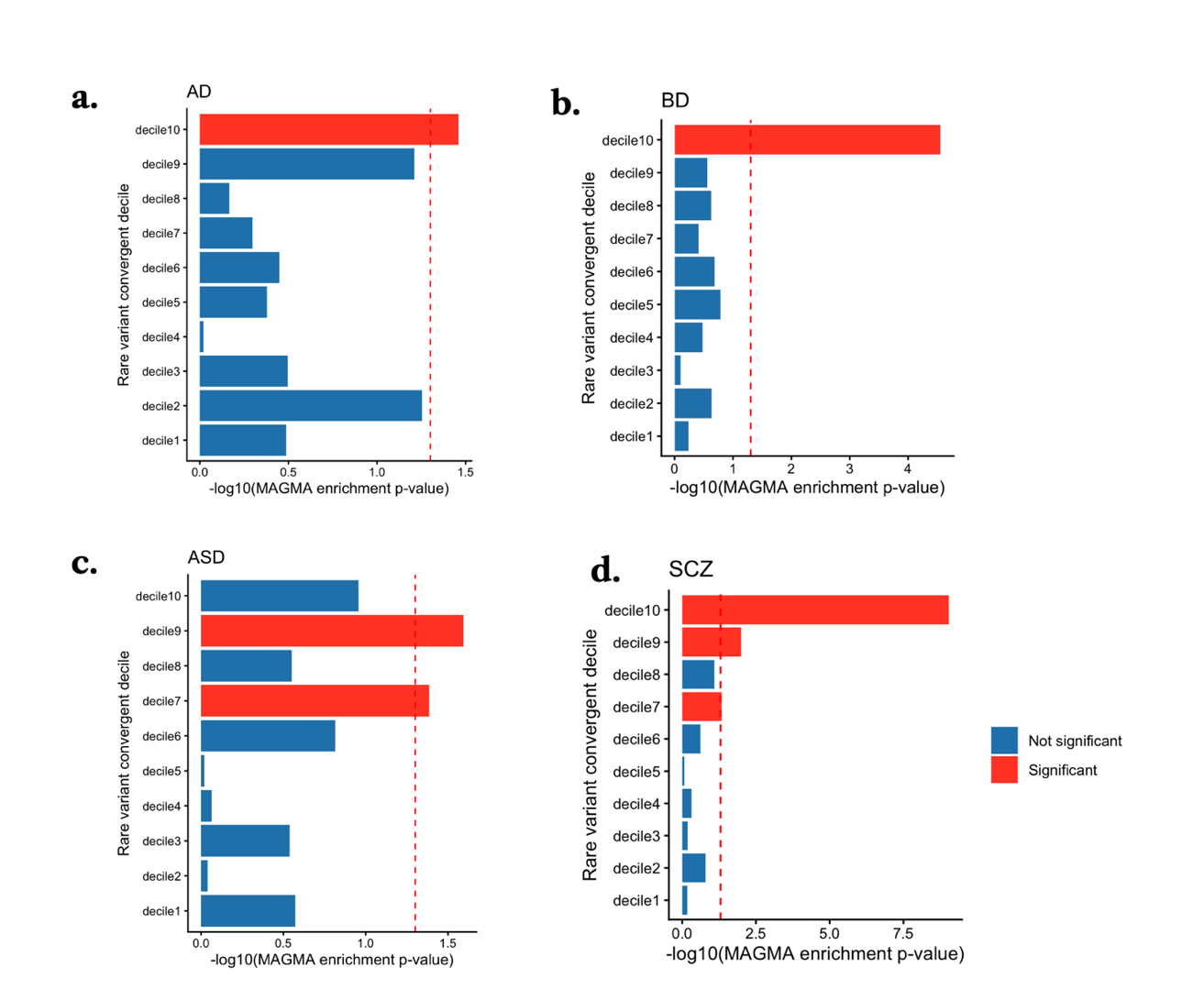

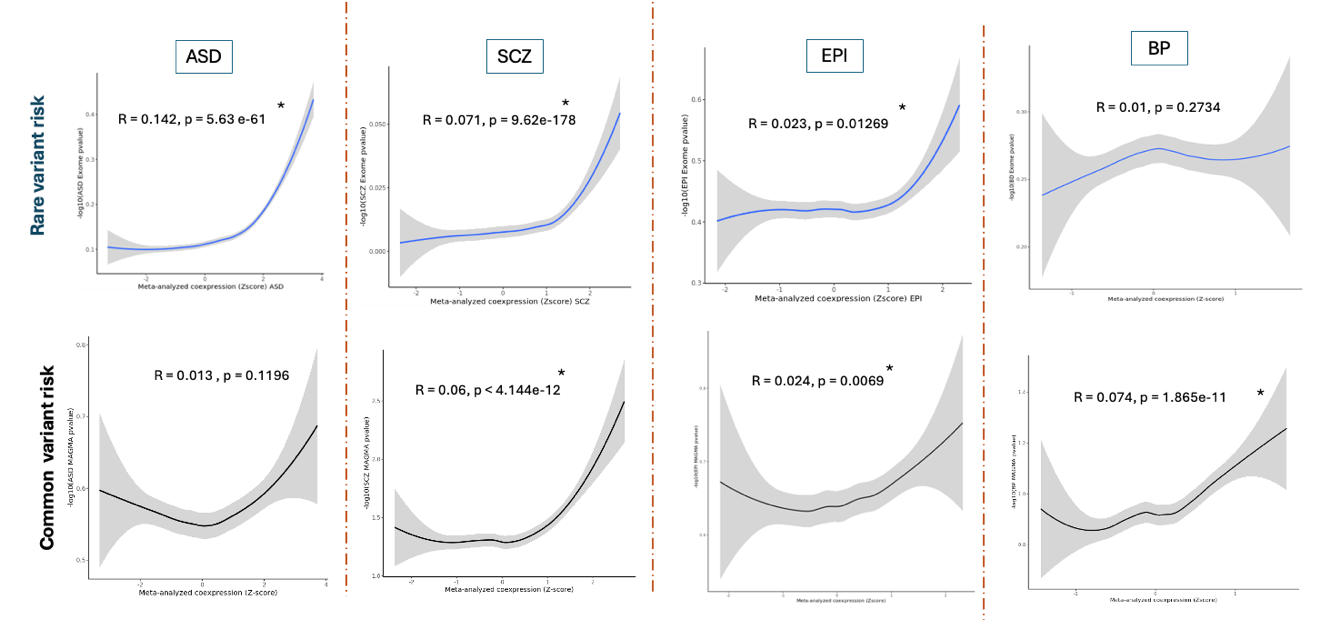


**e.**

**Figure S6.** A-D) MAGMA gene set enrichment output rare variant convergent genes. E) Correlation of burden convergence Zscores with exome and GWAS (MAGMA) association risk (pvalue).


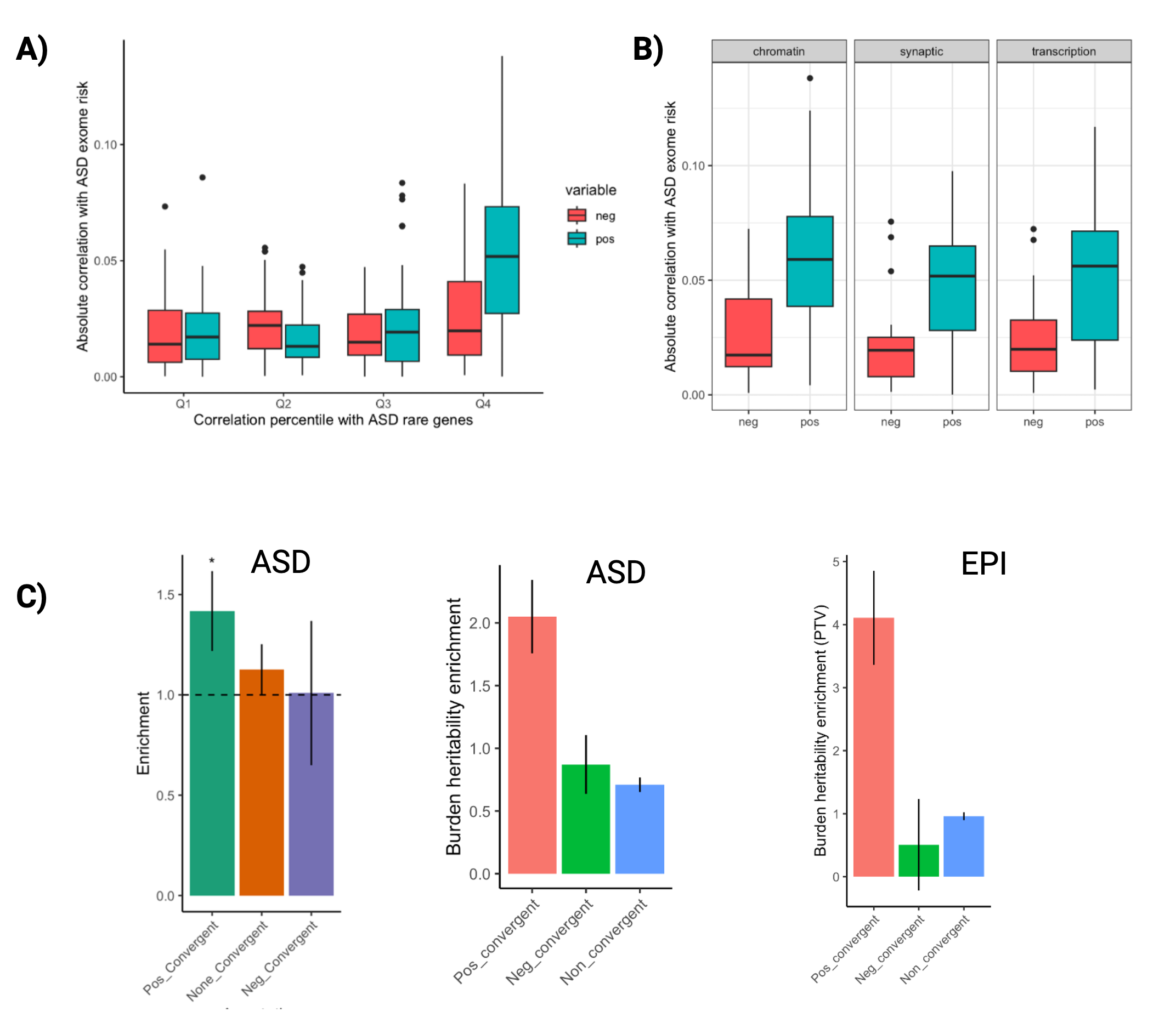


**Figure S7.** Stratification of Genes by Direction of Convergence Z-Scores

A) Correlation between burden convergence Z-score and exome association risk (p-value) for genes stratified by Z-score direction in ASD.

B) Correlation between burden convergence and exome p-value for genes grouped by functional categories and Z-score direction.

C) Per-SNP and burden heritability of significantly convergent genes (p.bonf < 0.05) in ASD and EPI, categorized by Z-score direction.


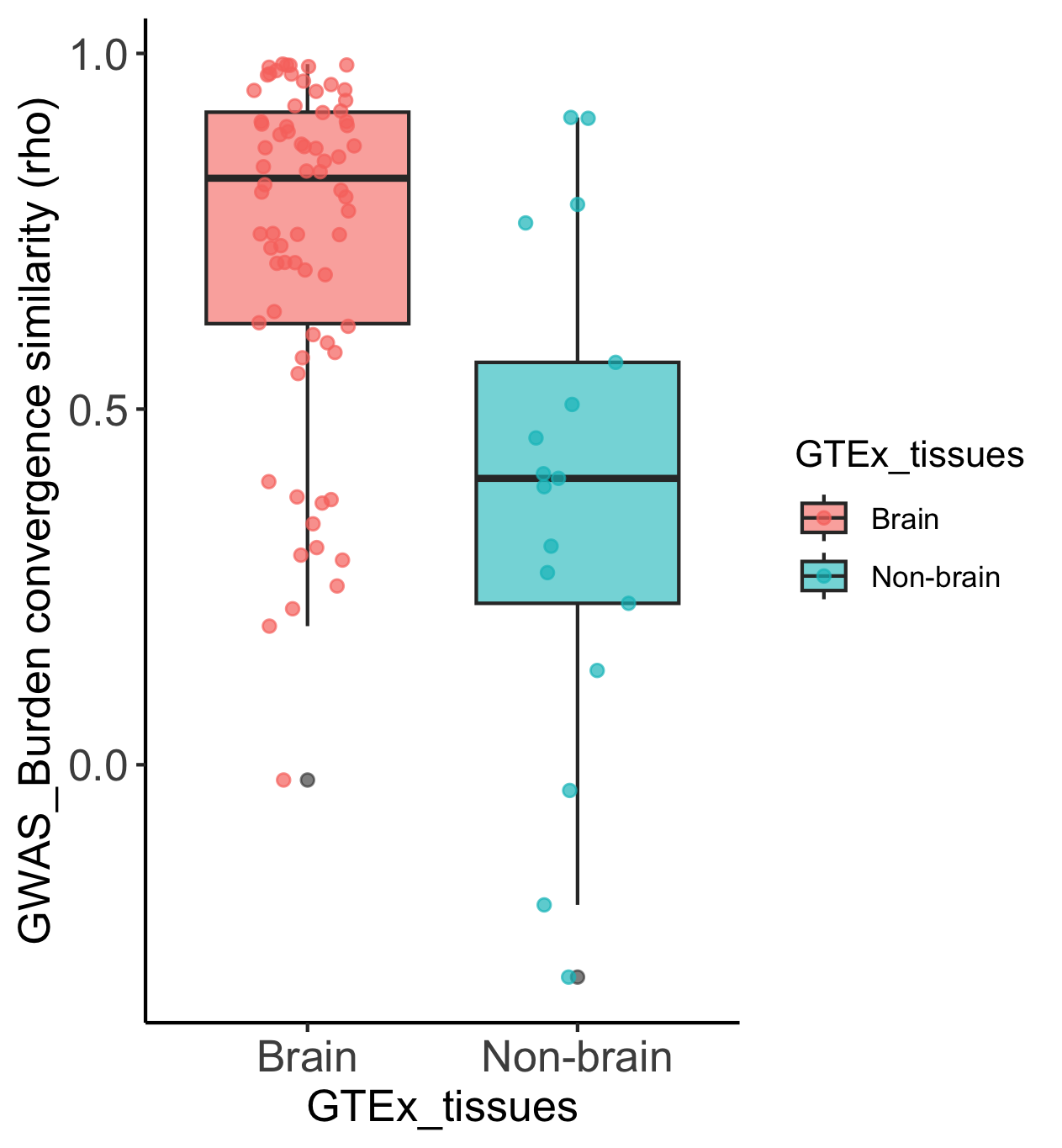

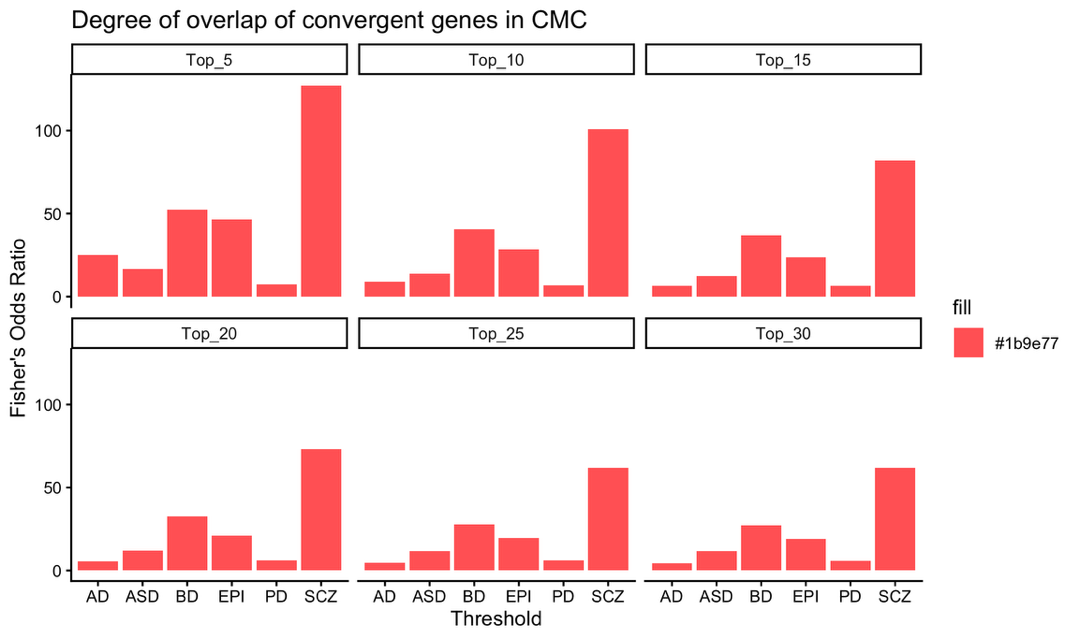


**A**

**B**


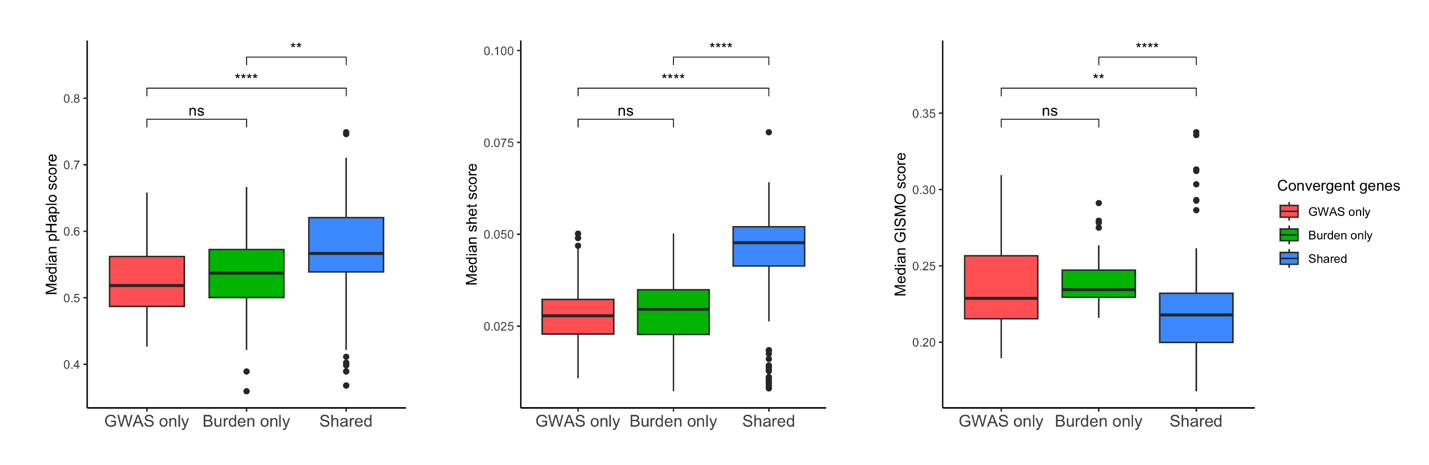


**C**

**Figure S8.** A) Similarity between GWAS and burden convergence Zscores in brain vs non brain tissues. B) Enrichment overlap between GWAS and burden convergent genes at different threshold cutoffs. C) Median intolerance (constraint) scores, including LOUEF, pHaplo, shet, and GISMO calculated for the top decile of GWAS only, Burden only, and shared convergent genes across six neuropsychiatric diseases, using coexpression data from 13 brain tissues. Significance of distributions is calculated using Wilcoxon rank sum *(***p < 0.001, p < 0.01, p < 0.05, ns = not significant.)* For shet and pHaplo, the higher the score, the more intolerant; for GISMO and LOEUF, lower scores indicate intolerance

**
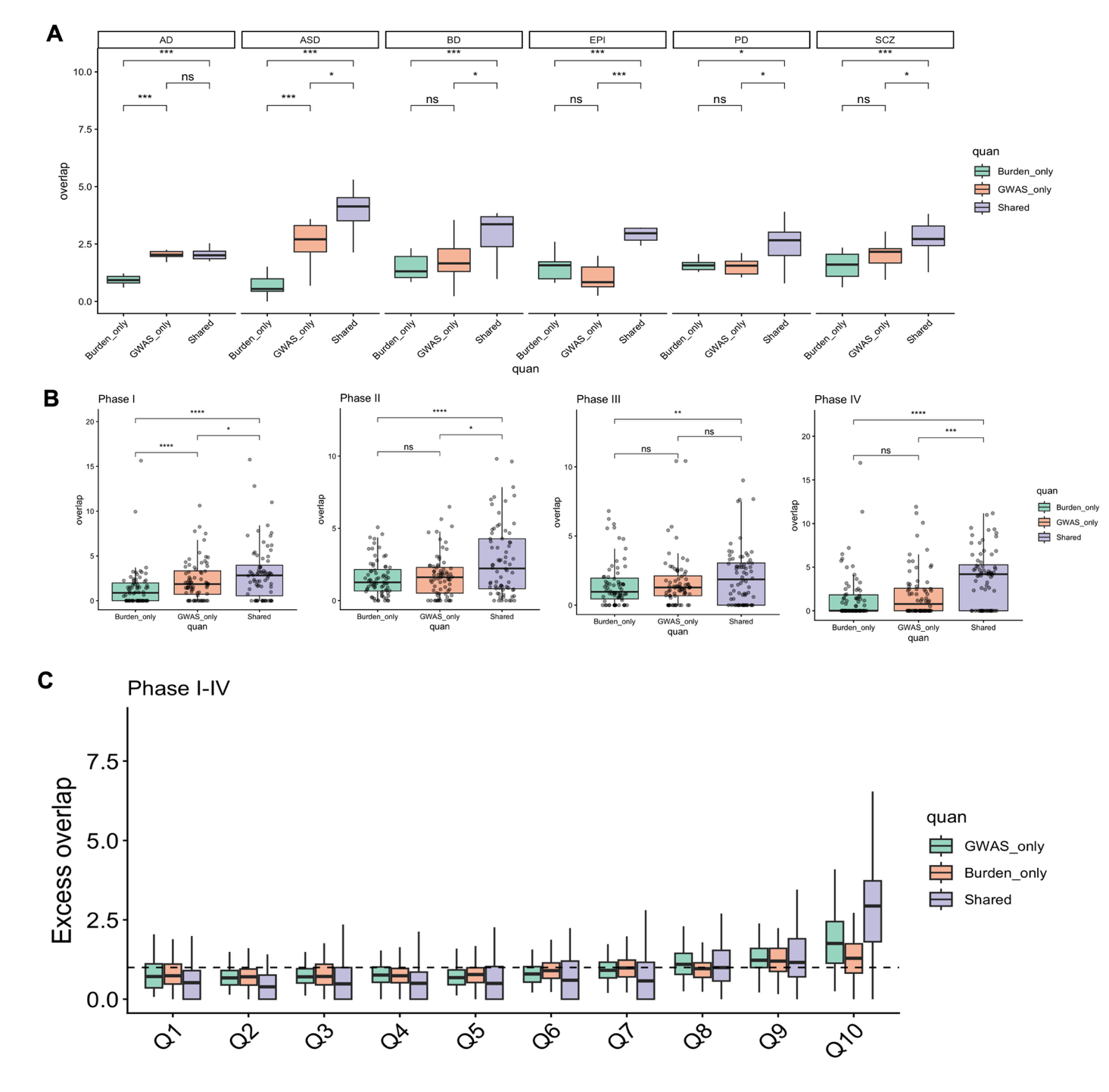
Figure S9.** Excess overlap of convergent genes with known drug targets (Open Targets) across burden-only, GWAS-only, and shared convergence sets. A) Excess overlap of top10 decile (> 90^th^ percentile) convergent genes with drug targets across all clinical phases and 13 brain tissues from GTEx and CMC; paired Wilcoxon rank was used for comparison, stratified by disorder. B) Excess overlap of known drug target genes at different clinical phases in convergent genes C) Excess overlap of drug targets across genes grouped by convergence deciles across the six disorders


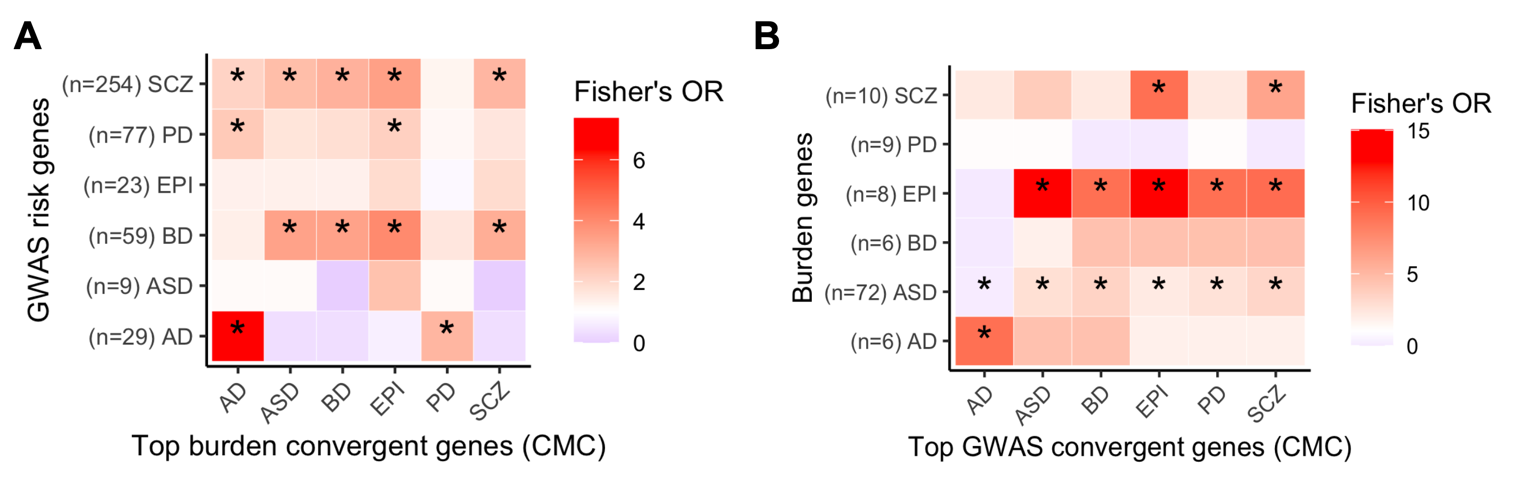


**Figure S10. A)** Enrichment of GWAS-prioritized risk genes among top rare variant burden convergent genes. The y-axis represents the number of prioritized GWAS risk genes. **B)** Enrichment of rare variant burden risk genes among top GWAS-convergent genes.
